# Case Report: A rare case of Epstein–Barr virus mucocutaneous ulcers occurring in the eyeball and literature review

**DOI:** 10.3389/fmed.2026.1749739

**Published:** 2026-02-10

**Authors:** Xiaokang Ke, Qingping Zhang, Wenxian Huang, Jie Rao, Jiacai Ren, Juan Wu, Honglin Yan, Jing Yuan, Jingping Yuan, Huihua He

**Affiliations:** 1 Department of Pathology, Renmin Hospital of Wuhan University, Wuhan, China; 2 Department of Radiology, Zhongnan Hospital of Wuhan University, Wuhan, China; 3 Department of Ophthalmology, Renmin Hospital of Wuhan University, Wuhan, China

**Keywords:** EBV, eyeball, keratoplasty, LPD, mucocutaneous ulcer

## Abstract

**Background:**

Epstein–Barr virus (EBV)-positive mucocutaneous ulcer (EBVMCU) is a shallow, sharply circumscribed, unifocal mucosal or cutaneous ulcer that often occurs in patients with immunosuppression.

**Case presentation:**

We report the case of a 79-year-old woman who underwent left eye keratoplasty for a corneal ulcer and continued topical immunosuppressive therapy after the operation, resulting in left eyeball lesions. Microscopically, the lesion is primarily located in the cornea and extends to the surrounding iris and choroidal tissues. Squamous epithelial ulceration and necrosis with exfoliation on the corneal surface and atypical lymphocytes of various sizes were observed, accompanied by dense polymorphic infiltration of various inflammatory cells, such as small lymphocytes, histiocytes, plasma cells, and eosinophils. These atypical cells had abundant cytoplasm with vesicular chromatin, distinct nucleoli, and scattered large cells resembling Hodgkin and Reed-Sternberg (HRS)-like cells. Immunohistochemistry and *in situ* hybridization (ISH) assays revealed that the atypical lymphocytes were positive for CD20, PAX5, CD30, MUM1, OCT-2, and EBV-encoded mRNA (EBER). Based on these findings, a diagnosis of EBVMCU of the left eyeball was rendered.

**Conclusion:**

To our knowledge, there are no reports of EBVMCU presenting as ocular lesions in the published literature. Therefore, understanding its specificity is very important for the correct diagnosis of this disease and to prevent misdiagnosis and mistreatment.

## Background

Epstein–Barr virus (EBV)-positive mucocutaneous ulcer (EBVMCU) was first described as a lymphoproliferative disorder (LPD) in 2010 when Dojcinov et al. (1) reported 26 patients with ulcerative lesions confined to the oropharynx, skin, and gastrointestinal tract. These lesions often occurred in patients with immunosuppression, including advanced age-associated immunosenescence, iatrogenic immunosuppression (1, 2), primary immune disorders (3), solid organ or bone marrow transplant recipients (4), and human immunodeficiency virus (HIV)/acquired immunodeficiency syndrome (AIDS)-associated immune deficiencies (5).

EBVMCU is a shallow, sharply circumscribed, unifocal mucosal or cutaneous ulcer that is histologically characterized by the proliferation of EBV-positive, variable-sized, atypical B-lymphocytes (6). These EBV-positive cells may resemble Hodgkin and Reed-Sternberg (HRS)-like or immunoblast-like cells, exhibiting a B-cell immunophenotype, such as a positive expression of CD20. Therefore, EBVMCU was initially misclassified as EBV-positive diffuse large B-cell lymphoma (EBV-positive DLBCL) or classical Hodgkin lymphoma (cHL) according to conventional histopathologic criteria (7). However, almost all patients with EBVMCU achieved spontaneous regression or completed remission even with reduced immunosuppressants and without chemotherapy, which differed from patients with EBV-associated lymphomas (6). Based on this, EBVMCU was first described as a rare provisional clinicopathological entity in the 2016 revision of the World Health Organization (WHO) classification of lymphoid neoplasms (8).

According to our literature review, a total of 186 cases of EBVMCU were reported from 2010 to 2020 (6). These lesions usually occur sequentially in the oropharynx, gastrointestinal tract, skin, palate, gingiva, esophagus, nasopharyngeal, and sinus. Recently, there was a case report of EBVMCU presenting as vulval introital ulcer (9). Until recently, there has been no report of EBVMCU presenting as ocular lesions in the published literature. In this study, we report a rare case of EBVMCU in the eyeball and thoroughly review the literature to summarize the clinicopathologic features of EBVMCU.

### Case presentation

**Figure 1:**
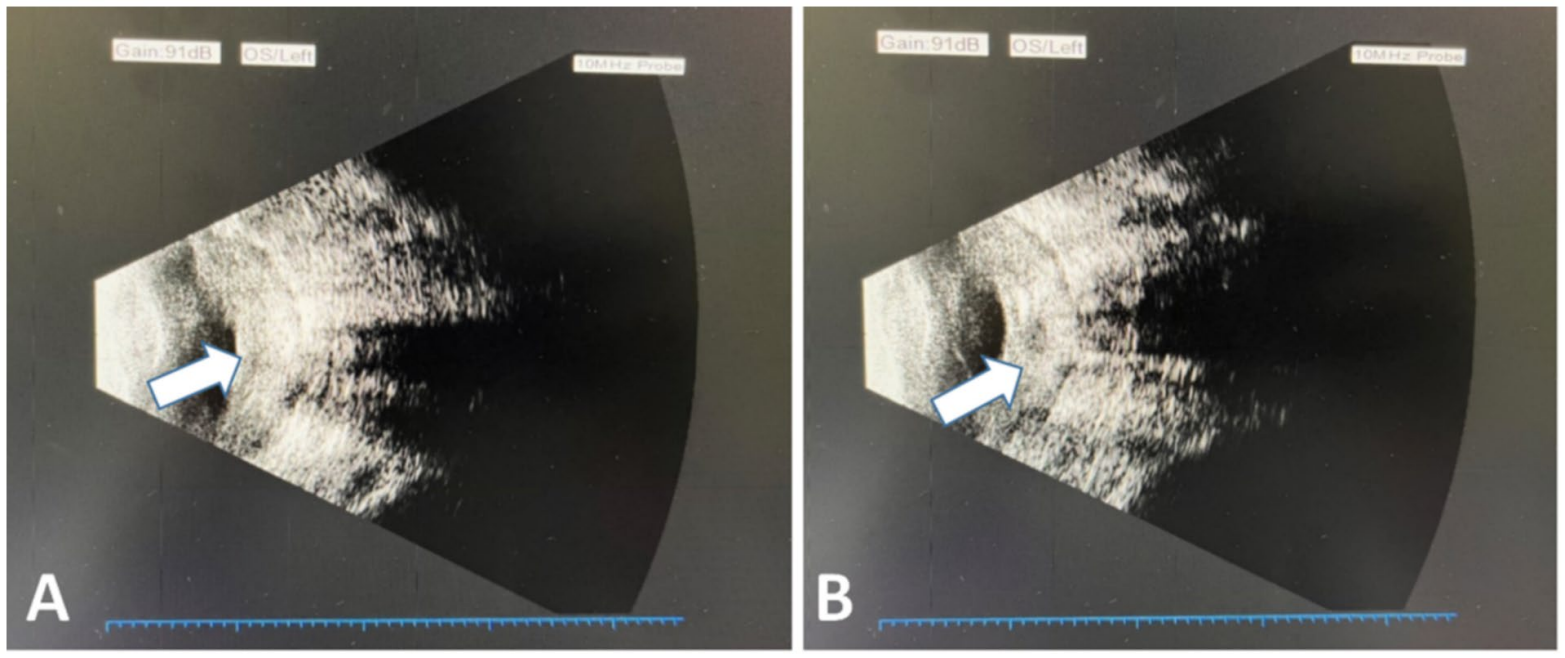
**(A, B)** B-scan ultrasonography demonstrating extensive vitreous opacities (white arrow).

We present the case of a 79-year-old woman who underwent left eye keratoplasty for a corneal ulcer more than 1 year earlier and remained on long-term topical medications, including tacrolimus (a topical calcineurin inhibitor) administered twice daily for approximately 1 year. During this period, she developed persistent discomfort in the left eye and was re-evaluated in the outpatient clinic. Ophthalmic examination showed no light perception in the left eye, and intraocular pressure could not be measured. The left conjunctiva was markedly hyperemic, and the cornea appeared reduced in size with yellowish opacity, precluding visualization of intraocular structures. B-scan ultrasonography demonstrated extensive vitreous opacities (Figure 1), raising concerns about endophthalmitis; therefore, evisceration was recommended to protect the contralateral eye by reducing the risk of sympathetic ophthalmia. There was no history of systemic immunosuppressive disease or systemic immunosuppressive therapy, apart from prolonged topical tacrolimus use following keratoplasty. HIV testing was negative. Complete blood count showed relative neutropenia (neutrophils 36.5%) and mild relative lymphocytosis (lymphocytes 52%), with the remaining parameters within normal limits. EBV DNA was undetectable in the peripheral blood. In July 2020, under local anesthesia of the patient’s left eye, a 360-degree limbal peritomy was performed, the bulbar conjunctiva was opened, the fibrous tissue was dissected, the cornea was excised approximately 2 mm posterior to the limbus, and the corneal specimen was sent with evisceration of the contents of the eye for histopathology. Histopathological examination of the lesion confirmed the diagnosis of EBVMCU. The patient subsequently tapered the dosage of tacrolimus and achieved complete remission after 3 months, with no evidence of disease at 18-month follow-up, as shown in Table 1.

**Table 1:** Timeline of diagnosis and management.

Time point	Key events	Diagnostic/Therapeutic actions
July 2019	A 79-year-old woman underwent left eye keratoplasty (due to corneal ulcer). Initiation of topical tacrolimus.	_
During this medication	Onset of eye discomfort	_
July 2020 (diagnosis)	Outpatient visit for discomfort	B-ultrasonography: large vitreous opacity. Blood test: EBV DNA not detected. Surgery and histopathology: corneal specimen examination.
		→ Definitive diagnosis: EBVMCU.
Post-diagnosis	Tacrolimus dosage tapered	_
After 3 months	Complete remission achieved	Clinical assessment.
~18 months	Disease-free at follow-up	Long-term monitoring.

### Histopathological findings

**Figure 2:**
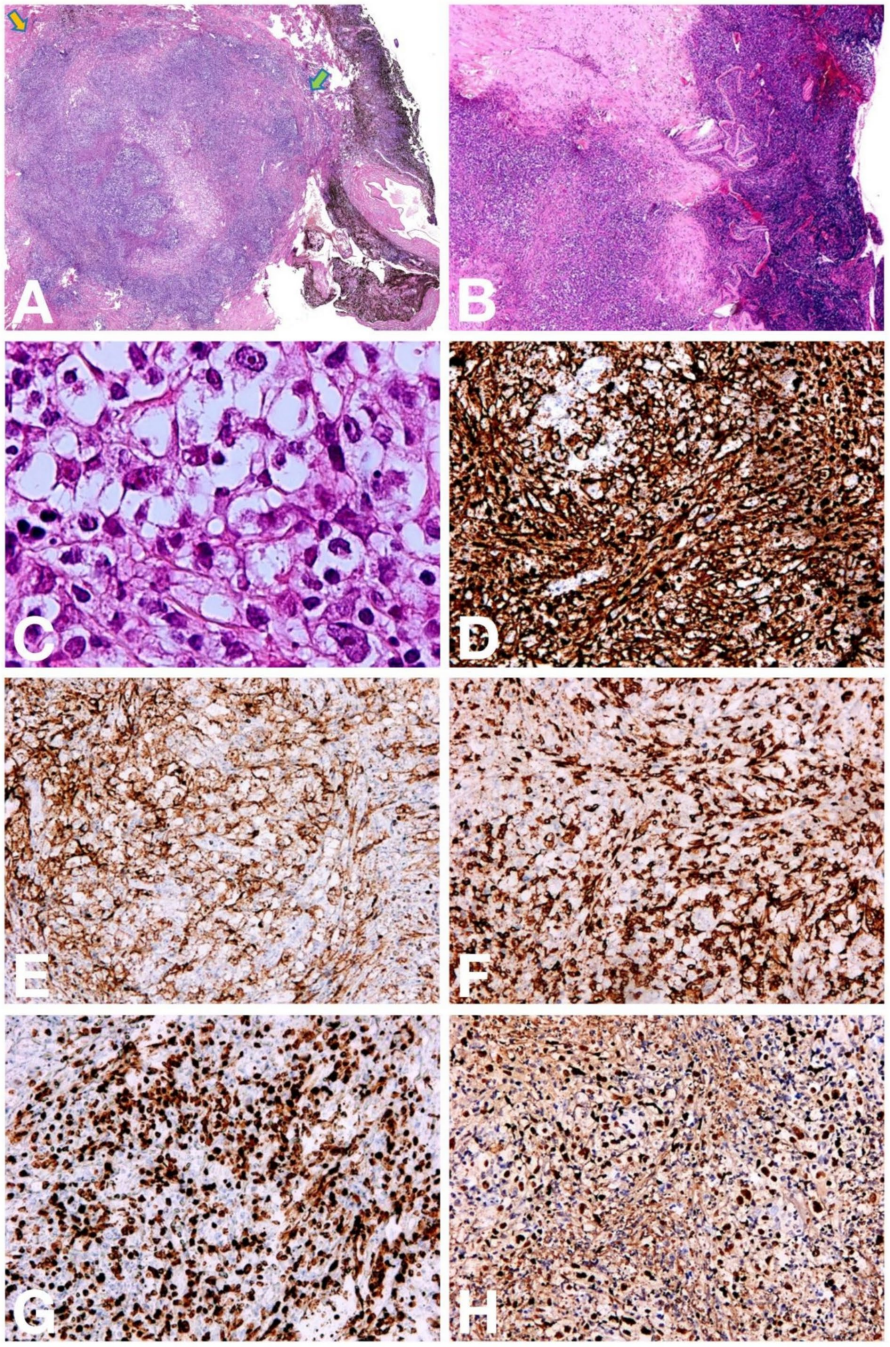
Pathologic findings of EBVMCU in the eyeball. **(A)** The lesion is primarily located in the cornea and extends to the surrounding iris and choroidal tissues. The yellow arrow indicates the anterior region of the cornea, while the green arrow denotes the posterior region of the cornea (×10). **(B)** Squamous epithelial ulceration and necrosis with exfoliation on the corneal surface, accompanied by a significant infiltration of atypical lymphocytes (×20). **(C)** These atypical cells had abundant cytoplasm with vesicular chromatin and distinct nucleoli, occasionally with a few HRS-like cells (×400). **(D,E)** Immunohistochemistry shows that these atypical cells were positive for CD20 (**D**, ×100) and CD30 (**E**, ×100). **(F)** Numerous CD3-positive T cells without atypia are observed in the background (×100). **(G)** The Ki67 proliferation index was approximately 50% (×100). **(H)** ISH assays show that atypical cells were positive for EBER (×100).

Microscopically, the lesion was primarily located in the cornea and extends to the surrounding iris and choroidal tissues (Figure 2A). Squamous epithelial ulceration and necrosis with exfoliation on the corneal surface and atypical lymphocytes of various sizes were observed (Figure 2B), accompanied by dense polymorphic infiltration of various inflammatory cells, such as small lymphocytes, histiocytes, plasma cells, and eosinophils. These atypical cells had abundant cytoplasm with vesicular chromatin, distinct nucleoli, and scattered large cells resembling Hodgkin and Reed-Sternberg (HRS)-like cells (Figure 2C).

### Immunophenotype and EBER *in situ* hybridization

Immunohistochemistry revealed that these atypical lymphocytes were positive for CD20 (Figure 2D), PAX5, CD30 (Figure 2E), MUM1, and OCT2 but negative for CD15, CD10, BCL-6, ALK, and C-myc. Numerous CD3 (Figure 2F), CD5, CD4, and CD8 positive T cells were observed in the background. The Ki67 proliferation index was approximately 50% (Figure 2G). Immunohistochemistry and *in situ* hybridization (ISH) for Epstein–Barr virus-encoded ribonucleic acid (EBER) were positive (Figure 2H). The findings supported the diagnosis of EBVMCU in the left eyeball.

### Molecular features

PCR analysis of both the immunoglobulin heavy chain (IgH) gene and T-cell receptor (TCR) gamma gene revealed a polyclonal background, suggesting that there was no clonal gene rearrangement pattern in this case.

## Discussion

EBV is a member of the herpes virus family that can cause latent infection in humans as well as B-cell proliferation and transformation (7). Therefore, even during the latency period, it may lead to various diseases, including lymphomas and LPD. EBVMCU is a recently recognized EBV-associated B cell LPD driven by latent EBV infection.

As a distinct clinicopathological entity, EBVMCU was first described by Dojcinov et al. (1) in 2010 and primarily occurs in immunosuppressed patients exhibiting either age-related immunosenescence or iatrogenic immunosuppression. Dojcinov et al. (1) reported a case series of 26 patients with EBV-associated B-cell LPD with well-defined ulcers involving the oropharynx, skin, and gastrointestinal tract. Twenty patients were followed up, and all patients achieved complete remission without disease-related death. In 2014, Hart et al. (4) identified 7 patients with EBVMCU among 70 solid organ transplant recipients with EBV-positive post-transplant lymphoproliferative disease (PTLD), including 5 renal transplants, 1 heart transplant, and 1 lung transplant. These seven patients received immunosuppressive therapy for 6 months to 13 years before the onset of ulceration, and all patients were negative for the EBV DNA test in their peripheral blood. Ulcers can be cured by adjusting the dose of immunosuppressants. Coincidentally, the clinical course of the case in this study is basically consistent with that reported in the above study. In 2015, Bunn and Van Heerden (5) reported two cases of AIDS-related EBV-MCU. Thus, EBVMCU primarily occurs in immunosuppressed patients, including those with advanced age-associated immunosenescence, iatrogenic immunosuppression (1, 2), primary immune disorders (3), solid organ or bone marrow transplant recipients (4), and HIV/AIDS-associated immune deficiencies (5).

According to our literature review, a total of 186 cases of EBVMCU were reported from 2010 to 2020 (6). In these case reports, the median age of patients with EBVMCU was 71 years (age range, 0.4–101 years), with a slight predominance of female patients. The majority of patients with EBVMCU exhibited indolent disease progression and spontaneous regression after discontinuation or dosage reduction of immunosuppressants, chemotherapy, or radiotherapy; however, a few reported cases had experienced recurrence (3) or even transformed into cHL (10). To date, EBVMCU has been reported predominantly in the oropharynx, gastrointestinal tract, skin, and other mucosal sites, whereas ocular involvement has not been previously described. In this study, we present a rare case of EBVMCU arising in the eyeball, which is worth reporting to the medical community.

EBVMCU is a shallow, sharply circumscribed, unifocal mucosal or cutaneous ulcer that is histologically characterized by the proliferation of EBV-positive, atypical B lymphocytes, accompanied by dense polymorphic infiltration of various inflammatory cells, such as plasma cells, histiocytes, and granulocytes. The atypical B cells vary in size from small to large and may resemble HRS-like cells or immunoblast-like cells. Angioinvasion and necrosis can be present in addition to surface ulceration. These overlapping features constitute a major diagnostic pitfall, as EBVMCU may be misinterpreted for aggressive EBV-associated lymphomas—most notably EBV-positive DLBCL (8) or cHL (7)—particularly in limited biopsies and in ocular specimens. Therefore, careful clinicopathological correlation is essential: recognition of a localized, sharply demarcated ulcerative process, confirmation by EBER *in situ* hybridization and appropriate immunophenotyping, and exclusion of systemic disease when clinically indicated (clinical assessment and staging when appropriate) can help avoid overdiagnosis and unnecessary systemic chemotherapy. Importantly, EBVMCU typically follows a localized and indolent clinical course, and the majority of cases improve with reduction of the immunosuppressive trigger and/or local measures (7, 8). In the present case, prolonged topical tacrolimus exposure supported the role of local immunosuppression, and tapering the agent was followed by complete remission.

A recent study by Ikeda et al. classified EBVMCU into the following four morphological subtypes based on histological features: (1) polymorphous, (2) large cell-rich, (3) cHL-like, and (4) mucosa-associated lymphoid tissue (MALT) lymphoma-like (2). In the polymorphous subtype, polymorphous refers to cases with various small-to-large atypical EBV-positive lymphocytes, occasionally with a few HRS-like cells. Atypical lymphocytes are densely clustered or scattered. In the study by Ikeda et al., 59% of cases were classified as the polymorphous subtype (2). In the large cell-rich subtype, a dense proliferation of large, monomorphic, atypical EBV-positive lymphocytes closely resembles DLBCL, and 21% of cases belonged to the large cell-rich subtype in the study by Ikeda et al. In the cHL-like subtype, numerous HRS-like EBV-positive cells and various sizes of EBV-positive atypical lymphocytes are observed, sometimes accompanied by epithelioid granulomas and eosinophilic infiltration. The aggregation of CD30-positive HRS-like cells is highlighted, similar to those in cHL. In the study by Ikeda et al. (2, 6), 12% of cases belonged to the cHL-like subtype. The final subtype is the MALT lymphoma-like subtype, in which small-to-medium-sized atypical lymphocytes with centrocytic-like features and/or plasmacytic features proliferate within the expanded interfollicular zone. In the study by Ikeda et al., 9% of cases were classified as this subtype.

In most cases of EBVMCU, EBV-positive cells are positive for CD20 and CD30 and exhibit characteristics of activated B lymphocytes. These cells are also usually positive for CD79a, PAX5, OCT2, and MUM1, with variable expression of BOB1. CD15 is expressed in approximately 50% of cases (6, 7, 10). Nearly one-third of cases exhibit IgH gene rearrangements and TCR gene rearrangements (6, 7). Thus, IgH rearrangements are not useful for distinguishing between EBVMCU and DLBCL. Ibrahim et al. (11) suggested that TCR gene rearrangements could lead to a limited T cell repertoire, which triggers EBV infections in the elderly and immunocompromised patients. This may eventually promote the formation of EBVMCU.

The correct diagnosis of EBVMCU based on its specific morphological subtype is extremely challenging, so the case in this study requires differential diagnosis of EBV-positive DLBCL, cHL, and EBV-positive marginal zone lymphoma (MZL). EBV-positive DLBCL is an aggressive B-cell lymphoma associated with chronic EBV infection and has a poor prognosis with standard chemotherapy (12). EBV-positive DLBCL was recognized as a clinicopathological entity in the 2016 revision of the WHO classification of lymphoid neoplasms, which highlights that the disease frequently affects both young and elderly immunocompetent patients (8). Because the pathological morphology and immunophenotype of EBV-positive DLBCL are not significantly different from EBVMCU, and IgH rearrangement is not useful for distinguishing between the two diseases, it is extremely difficult to distinguish between the two. However, EBVMCUs typically have “localized lesions.” In addition, clinical manifestations are also necessary to be considered when distinguishing between the two diseases. EBV-positive DLBCL usually presents an aggressive clinical course with frequent extranodal disease. Conversely, EBVMCU is an ulcerative EBV-positive B cell LPD with a self-limited indolent course. A recent study found that PD-L1 may be a new distinguishing point between EBV-positive DLBCL and EBVMCU (13). This study showed that PD-L1 was present in the majority of EBV-positive DLBCL cases but was absent in almost all cases of EBVMCU (13).

The second differential diagnosis to be considered is cHL. Although the histological features of cHL-like EBVMCU are similar to cHL, many of the B lymphocytes are CD20-negative, which is present in cHL. In addition, patients with cHL rarely develop extranodal lesions. Therefore, cHL and EBVMCU can be distinguished according to their respective clinicopathological features (7).

Another disease for differential diagnosis is EBV-positive MZL. Several reports have shown that EBV-positive MZL occurred in immunosuppressed patients (2, 14). These reports have found that the majority of patients had a clinically indolent disease that responded to reduced immunosuppression. Furthermore, the clinical presentations differ between MALT lymphoma-like EBVMCU and EBV-positive MZL. For example, in previous reports, EBV-positive MZL was described as non-ulcerative neoplastic lesions outside the oral cavity, whereas MALT lymphoma-like EBVMCU was characterized by oral and ulcerative lesions. Considering the clinical presentation, MALT lymphoma-like EBVMCU differs from EBV-positive MZL.

## Conclusion

In conclusion, we describe a rare ocular case of EBVMCU. EBVMCU is a localized, sharply circumscribed ulcerative EBV-driven B-cell lymphoproliferative disorder that typically arises in the setting of immunosuppression. In clinical practice, EBVMCU should be considered in a unifocal ocular ulcerative or mass-forming lesion, particularly in the context of local immunosuppression, such as prolonged use of topical calcineurin inhibitors. Confirmation with appropriate pathology, including EBER *in situ* hybridization and immunophenotyping, is essential. Importantly, EBVMCU can closely mimic EBV-associated lymphomas such as EBV-positive DLBCL or cHL, and misclassification may lead to unnecessary systemic chemotherapy. Recognition of EBVMCU and careful clinicopathological correlation help guide appropriate management—often involving the reduction of immunosuppression and local therapy—and can prevent overtreatment.

## Data Availability

The original contributions presented in the study are included in the article/supplementary material, further inquiries can be directed to the corresponding authors.

## References

[1] Dojcinov SD Venkataraman G Raffeld M Pittaluga S Jaffe ES. EBV positive mucocutaneous ulcer--a study of 26 cases associated with various sources of immunosuppression. Am J Surg Pathol. (2010) 34:405–17. doi: 10.1097/PAS.0b013e3181cf8622, 20154586 PMC6437677

[2] Ikeda T Gion Y Sakamoto M Tachibana T Nishikori A Nishimura MF . Clinicopathological analysis of 34 Japanese patients with EBV-positive mucocutaneous ulcer. Mod Pathol. (2020) 33:2437–48. doi: 10.1038/s41379-020-0599-8 32561847

[3] Natkunam Y Goodlad JR Chadburn A de Jong D Gratzinger D Chan JK . EBV-positive B-cell proliferations of varied malignant potential: 2015 SH/EAHP workshop report-part 1. Am J Clin Pathol. (2017) 147:129–52. doi: 10.1093/ajcp/aqw214, 28395107 PMC6248636

[4] Hart M Thakral B Yohe S Balfour HH Singh C Spears M . EBV-positive mucocutaneous ulcer in organ transplant recipients: a localized indolent posttransplant lymphoproliferative disorder. Am J Surg Pathol. (2014) 38:1522–9. doi: 10.1097/PAS.0000000000000282 25007145

[5] Bunn B Van Heerden W. EBV-positive mucocutaneous ulcer of the oral cavity associated with HIV/AIDS. Oral Surg Oral Med Oral Pathol Oral Radiol. (2015) 120:725–32. doi: 10.1016/j.oooo.2015.06.028, 26254983

[6] Ikeda T Gion Y Nishimura Y Nishimura MF Yoshino T Sato Y. Epstein–Barr virus-positive Mucocutaneous ulcer: a unique and curious disease entity. Int J Mol Sci. (2021) 22:1053. doi: 10.3390/ijms22031053 33494358 PMC7865427

[7] Ikeda T Gion Y Yoshino T Sato Y. A review of EBV-positive mucocutaneous ulcers focusing on clinical and pathological aspects. J Clin Exp Hematop. (2019) 59:64–71. doi: 10.3960/jslrt.18039 31257347 PMC6661964

[8] Swerdlow SH Campo E Pileri SA Harris NL Stein H Siebert R . The 2016 revision of the World Health Organization classification of lymphoid neoplasms—ScienceDirect. Blood. (2016) 127:2375–90. doi: 10.1182/blood-2016-01-643569 26980727 PMC4874220

[9] Ansar S Mahadik A Chow C Papapetros I Lee CS. Epstein-Barr virus positive mucocutaneous ulcer of vulva. Pathology. (2019) 51:543–4. doi: 10.1016/j.pathol.2019.03.004, 31221432

[10] Moran NR Webster B Lee KM Trotman J Kwan YL Napoli J . Epstein Barr virus-positive mucocutaneous ulcer of the colon associated Hodgkin lymphoma in Crohn's disease. World J Gastroenterol. (2015) 21:6072–6. doi: 10.3748/wjg.v21.i19.6072 26019475 PMC4438045

[11] Ibrahim HA Menasce LP Pomplun S Burke M Bower M Naresh KN. Presence of monoclonal T-cell populations in B-cell post-transplant lymphoproliferative disorders. Mod Pathol. (2011) 24:232–40. doi: 10.1038/modpathol.2010.186 20834235

[12] Castillo JJ Beltran BE Miranda RN Young KH Chavez JC Sotomayor EM. EBV-positive diffuse large B-cell lymphoma, not otherwise specified: 2018 update on diagnosis, risk-stratification and management. Am J Hematol. (2018) 93:953–62. doi: 10.1002/ajh.25112, 29984868

[13] Sakakibara AKK Ishikawa E Suzuki Y Shimada S Eladl AE Elsayed AA . Age-related EBV-associated B-cell lymphoproliferative disorders and other EBV + lymphoproliferative diseases: new insights into immune escape and immunodeficiency through staining with anti-PD-L1 antibody clone SP142. Pathol Int. (2020) 70:481–92. doi: 10.1111/pin.12946 32367595

[14] Gong S Crane GM McCall CM Xiao W Ganapathi KA Cuka N . Expanding the spectrum of EBV-positive marginal zone lymphomas. Am J Surg Pathol. (2018) 42:1306–16. doi: 10.1097/PAS.0000000000001113 29957733 PMC6133753

